# At what risk? A research note on interviewer burden

**DOI:** 10.1186/s13104-024-06839-z

**Published:** 2024-07-05

**Authors:** Khadijah Melvin, Erin Sweeney, Katherine Buchman, Eboni Winford, Jessica Ansah, Sandra Wairimu, Will Martinez, Judson Laughter, Jennifer Jabson Tree

**Affiliations:** 1 University of Tennessee, Knoxville, TN USA; 2 Purdue University, West Lafayette, IN USA; 3 Cherokee Health Systems, Knoxville, TN USA; 4 A418 Bailey Education Complex, Knoxville, TN 37996-3442 USA

**Keywords:** Institutional racism, Interviewer burden, Respondent burden, FQHC, Qualitative interview, Public health critical race praxis

## Abstract

**Background:**

We report on our methodological experiences during an investigation of how institutional racism functions in healthcare. We found tension between balancing methodological rigor with the unanticipated consequence of interviewer burden.

**Methods:**

Semi-structured interviews were conducted with patients. Interviews were recorded, transcribed verbatim, and qualitatively analyzed using thematic content analysis. Interviewers also participated in weekly debriefing sessions and reported experiences with patients.

**Results:**

Interviewers repeatedly experienced negative encounters with white patients during interviews. Themes included privilege to avoid racism, denial of racism, non-verbal discomfort, falsely claiming Native identities, and intimidation. These experiences were most pronounced with Black interviewers.

**Discussion:**

Interviewer burden may need to be a consideration taken up in a variety of research contexts.

**Supplementary Information:**

The online version contains supplementary material available at 10.1186/s13104-024-06839-z.

## Introduction

Federally Qualified Health Centers (FQHCs) provide care to those most in need in the United States; however, they are not immune from reproducing institutional racism. Our mixed methods project, guided by a Public Health Critical Race praxis [1] seeks to elucidate specific pathways perpetuating institutional racism and harming the health of Black, Indigenous, and People of Color (BIPoC). From this larger project, we report specific, unanticipated methodological consequences.

The larger project is a multi-level investigation of structural racism in a FQHC with the purpose of identifying policy and other mechanisms promoting anti-racism in healthcare. This involved policy analysis, semi-structured qualitative interviews with BIPoC and white patients and employees, quantitative surveys concerning FQHC climate, and patient health outcomes. While completing the qualitative dimension of this larger project, we conducted interviews with white patients about their observations and experiences with structural racism. This data collection led to *interviewer burden*; this concept extends the well-established ethical concern of *respondent burden* [2]. The purpose of this research note is to discuss the concept of interviewer burden informed by interviewer experiences, team response, and necessary methodological changes to the project.

## Research context and theoretical framing

FQHCs were initially designed during Lyndon Johnson’s *War on Poverty* in 1965 [3] to provide safety-net outpatient services to the United States’ most vulnerable citizens [4]; they now exist in every state and territory. As with all healthcare institutions in a country grounded in societal racism [5], FQHC policies, practices, operations, and functions may unintentionally reflect the influences of racism. This was illustrated by Warner et al. [6], who demonstrated that social contexts existing externally of a healthcare center directly influence the availability and quality of health services provided by FQHCs. Likewise, Lee at al. [7] demonstrated how regional context influences care utilization at FQHCs, while Snowden et al. [8] reported that regions with high racial bias had fewer FQHCs.

As FQHCs are not immune to the presence and effects of structural racism, our purpose was to apply Public Health Critical Race praxis (PHCR [1]) to elucidate and describe specific policy and other pathways, informed by patient and employee experiences, that perpetuate institutional racism in a FQHC in a midsized Southeastern city. This work is necessary because without knowing the systems or programs that manifest and reproduce institutional racism in FQHCs, it is not possible to identify areas within the institution that require intervention.

PHCR praxis is a framework that centers racial justice and names 10 key concepts that underpin existence and experiences of structural racism. These concepts are presented and defined in Table 1. For example, “ordinariness of racism” represents the idea that racism is inextricably embedded in all levels of our social fabric. The ordinariness of racism renders it cannot be seen except when it occurs in discreet and overtly egregious individual actions. Ordinariness does not seek to negate the presence of racism; rather it communicates how racism is so pervasive as to remain largely unseen [1].

**Table 1:** Public health critical race praxis (PHCRP) principles and definitions

Principle	Definition
Race consciousness	Deep awareness of one’s racial position; awareness of racial stratification processes operating in colorblind contexts
Primacy of racialization	The fundamental contribution of racial stratification to societal problems; the central focus of CRT scholarship on explaining racial phenomena
Race as a social construct	Significance that derives from social, political, and historical forces
Ordinariness of racism	Racism is embedded in the social fabric of society
Structural determinism	The fundamental role of macro-level forces in driving and sustaining inequities across time and contexts; the tendency of dominant group members and institutions to make decisions or take actions that preserve existing power hierarchies
Social construction of knowledge	The claim that established knowledge within a discipline can be re-evaluated using antiracism modes of analysis
Critical approaches	To dig beneath the surface; to develop a comprehensive approach
Intersectionality	The interlocking nature of co-occurring social categories (e.g., race and gender) and the forms of social stratification that maintain them
Disciplinary critique	The systematic examination by members of a discipline of its conventions and impacts on the broader society
Voice	Prioritizing the perspectives of marginalized persons; Privileging the experiential knowledge of outsiders within
Adapted from Ford and Airhihenbuwa, 2010	

Conventional research methods apply several strategies in the name of scientific rigor, including inter-group comparisons where one group is considered a norm against which other groups are measured. Historically, the normative group in health services research been and remains white patients, reinscribing the idea that white patients are the comparative research standard. Therefore, when our larger project was initially conceptualized, white patients were included in the recruitment frame as participants.

As acknowledged by PHCR praxis, racism influences all patients’ lives and experiences, including white patients. Although perhaps outside their awareness and desire, white patients often benefit from institutional racism, experiencing better access to healthcare services, higher quality care, healthier outcomes, and longer lives. They may also experience deleterious effects of racism, such as not having necessary FQHC availability [7, 8]. Finally, it is possible that white patients observe or experience racism while in healthcare settings. Understanding these facets of racism was important to our project; therefore, white patients were recruited to provide patient perspectives on institutional racism.

Respondent burden is a common concern in health research and involves ethical consideration of “the perception by the participant of the psychological, physical, and/or economic hardship associated with participating in the research process.” [2] While conducting interviews with white patients, we encountered a phenomenon that extends this concept [2] to *interviewer burden* [9]. Japec [10] defined interviewer burden as “the total amount of perceived effort, both physical and cognitive, that an interviewer has to exert to complete an interview according to specifications.” Interviewer burden can also include any psychological or other hardship caused by conducting the interviews.

Interviewer burden has been considered previously in cases where interviewers can become traumatized by conducting clinical interviews with people surviving traumatic life experiences (e.g., intimate partner violence) [9]. However, additional factors could cause interviewer burden. According to Japec’s interviewer burden model [10], interviewer burden is produced by five factors: social environment, interviewer characteristics (e.g., training, demographic characteristics), tasks required of the interviewer, respondent characteristics, and interview method. Interviewer burden may be more likely to occur when interviewers conduct interviews concerning issues and stressors that they have personally experienced [9], as may be the case when doing interviews about causes of health disparities.

In the case of institutional racism, very little published evidence addresses interviewer burden among individuals who hold marginalized social positions (e.g., Black women) and engage in research activities that could expose them to additional racial and gendered forms of discrimination. The purpose of this research note is to describe an example of interviewer burden and the interviewer-centered processes used to modify methodologies to resolve interviewer burden and protect interviewer safety.

## Methods

A more comprehensive description of the main project’s methodological design is provided in forthcoming articles.

### Participants

This research note reports exclusively on interviewer experiences interviewing white patient participants. Interviewers were master’s and doctoral students in public health and were employed by the larger research project. Interviewers were selected based on their documented training in institutional racism, PHCR praxis, qualitative interviewing, and health disparities. Interviewers represented diverse racial and ethnic identities, genders, and sexual orientations; four interviewers were Black, three were white, three identified as queer, and one identified as gender-queer.

Recruitment procedures for the full project are reported in a forthcoming manuscript. Briefly, all patient participants were recruited from a FQHC located in a mid-sized city in the Southeastern United States. Patient participants were recruited by trained interviewers who distributed hard copy recruitment fliers weekly, in person, to patients. Participants were eligible for participation if they had received care from the FQHC within the past 12 months and could speak and understand English well enough to participate in the interview. Participants were compensated with $50 gift cards for interview participation. BIPoC and white patients were recruited for participation, but only white patient data are presented here. BIPoC patient data will be presented in a forthcoming manuscript.

### Data collection

Interviewers conducted semi-structured, qualitative interviews with eligible participants. Interviewers were trained by the project’s leadership and in graduate coursework in the PHCR praxis, qualitative interviewing techniques, and the interview guide. All interviewers conducted pilot interviews to demonstrate proficiency with the interview guide and concepts. Pilot interviews were reviewed and critiqued by project leadership prior to initiating data collection. Interviewers and interviewees were not initially matched by race/ethnicity.

The interview guide was informed by PHCR praxis and included questions about institutional racism observed and experienced at the FQHC; the interview guide developed for this study is included as supplementary material. Interviews were audio-recorded for transcription and analysis. Transcription was verbatim and completed by a professional transcription service.

### Qualitative analyses

Qualitative analyses of interviewer experiences with white participants were conducted during weekly 90-minute team debriefing sessions and discussions with interviewers. These sessions included in-depth discussion about each interview and its process. Full qualitative analyses with the transcripts produced by the larger project involved thematic and content analyses applying a deductive codebook based on the PHCR praxis and microaggressions, as defined by Sue and colleagues [11]. These qualitative results are reported in a forthcoming manuscript.

The qualitative analysis for this research note involved in-depth discussions with the full interviewer team regarding negative experiences reported by multiple interviewers. Over three weeks and across multiple interviews, interviewers described experiencing negative incidents across multiple patient participants. During the debriefing sessions, interviewers described and used transcripts to document extensive, negative experiences with white patient participants. These experiences were sorted into themes and used to inform rapid, safety-oriented methodological changes to the interview process. Recruitment and interview protocols were modified in three ways: (1) racial concordance between interviewers and interviewees (only white interviewers interviewed white patients), (2) all interviewers worked in pairs during all recruiting and interviewing, and (3) ultimately ending white patient participant recruitment.

## Results

Interviews with BIPoC patients (*N* = 33) yielded fruitful results on experiences of institutional racism including describing the “ordinariness of racism” specified by the PHCR praxis; BIPoC patients reporting experiences of racism in the FQHC emphasized the need for providers and staff to be educated about institutional racism across all levels, from system policies down to interpersonal interactions. A complete report of the results for these data is forthcoming.

Interviews conducted with white patients (N = 18) led to dangerous circumstances for interviewers and subsequent termination of further interviews with white patients, our example of *interviewer burden*. Interviewer burden was exemplified in three ways: 1. White patient participants communicated disinterest in and disregard for structural racism, represented by impatience and eagerness to end their interviews. 2. During the interviews, white participants repeatedly, and sometimes urgently, asked about compensation. 3. When interviewers followed the interview guide as directed by the protocol, several white participants committed racial microaggressions against Black interviewers, others became openly angry and intimidating toward interviewers. Experiences reported by interviewers represented two themes: feelings of discomfort and fear and feelings of concern for interviewer team safety and psychological harm. During multiple interview incidents, interviewers reported feeling uncomfortable, scared, overwhelmed, and concerned for their own, and their colleagues’, safety. When Black interviewers interviewed white participants, the intimidation was more pronounced.

For example, one interviewer, a Black woman, documented her experience with a white participant:

When I asked questions about the [white] patient’s experience with racism, he stated it was “going to become more of a thing of the past.” He went on to say that racism was a result of being “uneducated and countryfied.” Throughout the interview, he urged me to continue by using phrases like, “I’ll let you go on and ask more questions” or “I’ll let you go ahead.” He described that he had “Indian” heritage [referring to Indigenous ancestry] and stated, “my sister is about as dark as you. I don’t know if you’re Indian. You got part Indian, or something?” This made me feel extremely uncomfortable–having my own racial/ethnic identity questioned. Despite feelings of discomfort, I continued. Near the end of the interview, we discussed feelings about racial concordance and the patient seemed to become agitated. He stated, “I don’t even think about that, ma’am. I think that’s foolish to even try to get into and look at it like that. It doesn’t matter to me if they’re Black, White, Pink, Yellow, or whatever.” I was sensitive to his agitation and decided not to ask any probing questions about those statements out of fear of further agitating the interviewee and escalating the situation.

As interviewing continued over time, the interview team repeatedly expressed concern that negative experiences, particularly for Black interviewers, added unjust psychological burden to their lived experiences with racism.

Consequently, the team agreed that perpetuating the risk of exposure to toxic interpersonal racism through Black interviewers interviewing white patient participants, on top of daily lived experiences with racism, was unethical. Thus, we decided that only white interviewers would interview white patient participants. Unfortunately, this strategy failed to resolve our safety issues. One white queer interviewer described a dangerous incident:

During a recruiting and interviewing session, two white patients became agitated to the point of harassing me. Dissatisfied with having to wait their turn [to be interviewed and thus receive compensation], their behavior escalated while I was attempting to complete interviews with other participants. These two patients became so frustrated and angry with me that they started demanding gift cards. I felt intimidated by their behavior, raised voices, and agitation, and eventually left the recruiting site before attempting to interview these patients.

Such interactions represented the norm rather than the exception with white patient participants. Therefore, after many hours and several weeks of debriefing uncomfortable and sometimes threatening encounters, and only 18 interviews with white patient participants, we collectively decided to stop enrolling white patient participants for the safety of the team.

## Discussion

*Respondent burden* is a well-established and acknowledged concept in research ethics, defined by the World Medical Association [2]. *Interviewer burden* describes the psychological stress, physical burdens, and sany other hardship experienced when conducting interviews [12, 9, 10] This experience may be especially poignant when interviews focus on sensitive or traumatic experiences had by interviewees or interviewers. Reactions might include feelings of guilt, vulnerability, stress, and exhaustion [9, 13] and may be particularly acute for interviewers who hold minoritized identities.

We have presented extended examples of interviewer burden arising from a study concerning structural racism. We observed several of Japec’s five factors influencing interviewer burden including, social context, types of questions asked of interviewees, and interviewer characteristics [10]. In terms of social context, the FQHC is in the southeastern United States, a region characterized by strong racialization and social norms that resist acknowledging the existence of white supremacy and structural racism [7]. In terms of social context, white patient participants from this region may be socialized to be unaware of and resist the realities of structural racism in healthcare. In terms of types of questions, it is possible that the questions concerning observations and experiences with structural racism may have exposed white respondents’ attitudes and beliefs that deny or minimize the existence of structural racism. Finally, it is possible that disregard for an interviewer’s characteristics (e.g., Black women) may have allowed white participants to feel safe or justified in expressing their agitation. Together, these factors caused interviewer burden.

Interviewer burden was addressed with several specific approaches including: identifying risk among interviewers, providing interviewer support, conducting interviews in safe and secure environments, conducting interviews in teams, providing interviewers with time to debrief with interviewer peers and project leadership, making leadership available on-call for acute and urgent needs, holding regular team meetings, and prioritizing interviewer mental health. Notably, these strategies were applied because they exemplified the values that we hope to amplify in the FQHC. Upon review of the literature, we then observed that these strategies also align with those recommended by Urquiza et al. [9] This alignment between existing recommendations for addressing interviewer burden were previously unknown to us. This is powerful because it emphasizes the need to review and challenge research methodology that perpetuates harms such as systemic racism even in the absence of existing protocols regarding protections of not only human subjects but also of researchers.

Due to the close interpersonal relationships among team members fostered by weekly 90-minute team meetings, interviewer burden was identified quickly after the first interviews conducted with white patient participants. This facilitated burden monitoring, changes to research protocols, and prioritization of interviewer wellbeing. During the acute experiences, interviewers used a real-time emergency text thread to provide support, care, and solutions, looping in project leadership when needed. Project leadership offered individual and group support and debriefing during weekly team debriefing sessions.

Resolving these consequences required extensive resources in the form of emotional labor, detailed discussions, protocol adjustments, and time. The team’s decisions throughout the process were neither impulsive nor disconnected from empirical analysis, nor were they based on one or two incidents. When interviewer burden was identified, the interview protocol was changed in two ways that also align with recommendations offered by Urquiza and Japec [9, 10]. First, interviewers were required to work in teams of two. Second, interviewers were matched by racial identity with participants, such that white interviewers interviewed white patient participants. Unfortunately, these approaches did not resolve the growing interviewer burden and data collection with white patient participants had to be terminated to prioritize interviewer wellbeing.

We vehemently disagree with eliminating whole demographic groups from research inquiry due to researcher or interviewer discomfort. However, we learned from these experiences that there are times when teams must act responsibly and protect their physical and mental safety, even if it limits research findings. We reached this decision even before fully applying qualitative analysis to interviews with white patient participants. We determined that there was sufficient evidence that continuing with a particular protocol was harmful despite the possibility of additional data; this determination was independent of *code* or *meaning saturation* [14]. The team came to the consensus that any additional data collected with white patient participants were not worth the heightened risk of violence.

Institutional racism in healthcare is perpetuated by complicity and participation of people. Therefore, public health efforts to address institutional racism require engaging white participants, and this may be perceived as confrontational by people unaccustomed to examining their own role in or benefit from institutional racism. Innovative protocols and methods are recommended for ensuring high quality data collection while protecting research teams from experiencing unjust burden. Perhaps our experience of interviewer burden may stand as empirical evidence that only including white participants for comparison purposes is unnecessary and maintains white-supremacist assumptions in accepted methods. As our first experience of interviewer burden as justification for ceasing this line of data collection, we are unsure how to report these outcomes. Yet, in a world where white violence is increasingly seen as a justified response to discussions of racism, we hope our experiences inform others pursuing such necessary work.

All people are influenced by structural racism, irrespective of awareness; therefore, understanding white patients’ observations and experiences with structural racism may be useful in addressing structural racism in healthcare. Researchers and clinicians doing similar research in the future may be well advised to prepare systematic protocols informed by factors that increase interviewer burden [10] and use Urquiza’s recommendations for anticipating, identifying, and addressing interviewer burden [9]. Preparing such systematic protocols in advance of interviewer burden will better prioritize interviewer well-being, reduce interviewer burden, and strengthen scientific endeavor.

### Electronic supplementary material

Below is the link to the electronic supplementary material.

Supplementary Material 1

## Data Availability

Due the qualitative nature of this research, supporting data are not available. Participants of this study did not consent to their interview transcripts to be shared publicly in their entirety. The authors confirm that any data supporting findings of this research are available within the article. The interview guide included as a supplementary material was developed for this study.
