# Supplementary material for: At what risk? A research note on interviewer burden
Source: BMC Res Notes. 2024 Jul 5;17:186. doi: 10.1186/s13104-024-06839-z (PMC11227223; doi:10.1186/s13104-024-06839-z)
Supplement: Supplementary file 1 — Supplementary Material 1 [file 13104_2024_6839_MOESM1_ESM.docx]

**Patient Interviews (drawing from** [***Racial Discrimination in Health Interview Project***](https://citeseerx.ist.psu.edu/viewdoc/download?doi=10.1.1.696.5295&rep=rep1&type=pdf)**)**

1. Introduction
   1. Start with an overview of purpose of interview
      1. *The main purpose of these interviews is to hear about any specific events where you believed you were treated unfairly or badly that you thought were due to racism when you were getting care for yourself and your family at CHS.*
      2. *The main purpose of these interviews is to understand your experience receiving care at CHS. We’ll ask you questions about your providers, the front office staff, and anyone else you may interact with while at a CHS office.*
   2. Provide definition of structural racism (we can decide which one we want to use)
   3. Provide information about link between structural racism and health outcomes
2. Questions
   1. Before I get into all of my interview questions, I’m very curious to hear from you about your experiences getting care at Cherokee. Can you tell me the story of your experiences at Cherokee?
   2. In your opinion, what does racism in healthcare look like to you?
   3. Tell me about a time in the past year when it was easy or hard to get an appointment with the provider you wanted to see.
      1. [Note to Interviewer: if individual tells a story about an easy time, after they are done, ask about a hard time. Same if they start with a hard example, then follow up to ask about the easy example]
   4. Could you please describe a time in the past year when someone at Cherokee has tried to convince you that you don’t need the appointment you want or need? This might include examples like trying to get a referral for a specialty provider outside of Cherokee.
   5. In your opinion, why do you think it was hard to get the referral? Why did the person try to convince you that you didn’t need the appointment? What would have been a better experience for you?
   6. Thinking back over the past year, what are some examples of when it was hard to reschedule an appointment that you needed to reschedule?
   7. In your opinion, why was it hard to reschedule the appointment? What happened to make that hard? What would have made it easier for you?

Now we are going to change gears a little bit and think about times when you may or may not have experienced other and specific experiences of structural racism. Remember that structural racism refers to racially adverse discriminatory practices, pathways, and policies within healthcare systems like Cherokee Health. It might look like an inability to get the care you need, see the providers you need to see, or getting substandard medical care.

- 1. Thinking back over the past year, can you tell me about or describe any experiences you have had with structural racism anywhere?
  2. Now, let’s think about Cherokee specifically. Thinking back over the past year, can you tell me about or describe any incidents of discrimination based on your race when you were getting care at CHS?

Thank you, that’s very helpful. There are other things that others have shared that could happen to you or your family that you might say are structural racism. We want to hear about these experiences as well. I am going to list some examples, please tell me if you have experienced these and if you have, please describe your experiences.

- 1. In the past year, at Cherokee, were you ever….
     1. …Not allowed to schedule an appointment that you wanted to schedule
     2. …Not allowed to get a referral that you needed/wanted
     3. …Not allowed to get tests, labs or exams you needed/wanted
     4. …Unable to pay for prescriptions you needed
     5. Did any provider refuse to give prescriptions for pain
     6. Did any provider refuse to give other prescriptions for other issues
     7. Did you experience transportation challenges getting to Cherokee for needed care
     8. Did you have a hard time getting supplies you needed for your health? Things like bandages, glucose test strips, or other things to help you take care of your health.
     9. Has anything else happened at Cherokee that you think could fall under the category of structural racism?
        1. For example have you seen posters, educational videos, or other pamphlets that you think may represent structural racism?
        2. Tell me about who have noticed about who is in charge in terms of race. For example, any one you may see in your visit such as your PCP, BHC, therapists, nurses, techs, building ground staff, and front desk staff.

Thank you for sharing those experiences with me. Now we are going to think about interpersonal racism. This is the type of racism that looks like discriminatory action or bias between to people, based on race.

- 1. Thinking back over the past year, can you tell me about or describe any experiences you have had with interpersonal racism anywhere?
  2. Now, let’s think about Cherokee specifically. Thinking back over the past year, can you tell me about or describe any incidents of discrimination based on your race when you were getting care at CHS?

In addition to these examples, some people have shared other things that could happen at Cherokee that you or your family may say was racist. We want to hear about these experiences as well. I am going to list some examples, please tell me if you have experienced these and if you have, please describe your experiences.

- 1. Has a **primary care provider, therapist, or staff member** ever:
     1. *Talked down to you*
        1. *If yes: Was this your Primary Care Provider, Therapist, or Staff*
        2. *If yes, please ask them to describe the example*
     2. *Displayed an “attitude” while serving you*
        1. *If yes: Was this your Primary Care Provider, Therapist, or Staff*
        2. *If yes, please ask them to describe the example*
     3. *Watched you more closely than others*
        1. *If yes: Was this your Primary Care Provider, Therapist, or Staff*
        2. *If yes, please ask them to describe the example*
     4. *Did not treat you with dignity and respect*
        1. *If yes: Was this your Primary Care Provider, Therapist, or Staff*
        2. *If yes, please ask them to describe the example*
     5. *Ignored you*
        1. *If yes: Was this your Primary Care Provider, Therapist, or Staff*
        2. *If yes, please ask them to describe the example*
     6. *Gave you poor service compared to other patients*
        1. *If yes: Was this your Primary Care Provider, Therapist, or Staff*
        2. *If yes, please ask them to describe the example*
     7. *Gave you poor service compared to other patients of a different race.*
        1. *If yes: Was this your Primary Care Provider, Therapist, or Staff*
        2. *If yes, please ask them to describe the example*
     8. *Used a racial slur*
        1. *If yes: Was this your Primary Care Provider, Therapist, or Staff*
        2. *If yes, please ask them to describe the example*
     9. *Acted as if they were afraid of you*
        1. *If yes: Was this your Primary Care Provider, Therapist, or Staff*
        2. *If yes, please ask them to describe the example*
     10. *Acted as if they thought you were not smart*
         1. *If yes: Was this your Primary Care Provider, Therapist, or Staff*
         2. *If yes, please ask them to describe the example*
     11. *Acted like they thought you were dishonest*
         1. *If yes: Was this your Primary Care Provider, Therapist, or Staff*
         2. *If yes, please ask them to describe the example*
     12. *Harassed you*
         1. *If yes: Was this your Primary Care Provider, Therapist, or Staff*
         2. *If yes, please ask them to describe the example*
  2. Thinking back to the time when you felt that you were treated differently because of your race while receiving care at CHS:
     1. *Did you complain about the event?*
     2. *If so, how did you complain (e.g., note, talk to staff member, ask to speak to supervisor, patient satisfaction survey)*
     3. *What was the result of that complaint?*
     4. *If you didn’t complain, why not? What do you think stopped you from doing so?*

Thank you for sharing your experiences with me. [If experienced negative examples….]And, I’m sorry, you shouldn’t have to experience these things.

We are nearly done, and you’re doing a great job. We have just a few more questions and then we’ll be done with the interview. Are you doing ok? Would you like a break?

Ok. Great, we’ll continue. Some of the next questions will seem like I’m repeating myself. This is because we want to know about specific experiences you may have had with the different doctors that you might see at Cherokee. First, I’ll some questions about your primary care doctor, and then about a therapist you might see. Sound ok?

- 1. Think back to interactions with your primary care doctor. When you share concerns with your PRIMARY CARE PROVIDER, are there times when you think they don’t hear you? Or take you seriously? What do you think causes them to not take you seriously?
  2. How important is it to you to have a PRIMARY CARE PROVIDER who looks like you? Who is the same race as you?

How does it feel when you are seen by a primary care provider who is the same race as you? Now I’m going to ask about some specific experiences you may have had with your therapist or BHC.

- 1. Think back to interactions with your therapist. When you share concerns with your therapist/BHC, are there times when you think they don’t hear you? Or take you seriously? What do you think causes them to not take you seriously?
  2. How important is it to you to have a therapist who looks like you? Who is the same race as you?
  3. How does it feel when you are seen by a therapist or BHC who looks like you?

Now I’m going to ask you some specific questions about your experiences with front office staff. Think back to your interactions with front office staff.

- 1. When you share concerns with them, are there times when you think they don’t here you? Or take you seriously? What do you think causes them to not take you seriously?
     1. How important is it to you to have front office staff who looks like you? Who is the same race as you?
     2. How does it feel when you are seen by front office staff who looks like you?

Now I’m going to ask you some specific questions about your experiences with nursing staff. Think back to your interactions with nurses.

- 1. When you share concerns with them, are there times when you think they don’t here you? Or take you seriously? What do you think causes them to not take you seriously?
     1. How important is it to you to have nurses who looks like you? Who is the same race as you?
     2. How does it feel when you are seen by nurses who looks like you?

Now I’m going to ask you some specific questions about your experiences with lab techs. Think back to your interactions with lab technicians

- 1. When you share concerns with them, are there times when you think they don’t here you? Or take you seriously? What do you think causes them to not take you seriously?
     1. How important is it to you to have lab techs who looks like you? Who is the same race as you?
     2. How does it feel when you are seen by lab techs who looks like you?

That is the last question on the interview.

- 1. Is there anything that we haven’t asked you that you’d like us to know?

Thank you so much for your time and sharing your experiences with us. If you have any questions in the future, or if you remember something you think is important for us to know, please contact us anytime at the number listed on the consent form.
